# Inappropriate direct oral anticoagulant prescriptions in patients with non-valvular atrial fibrillation: cross-sectional analysis of the French CACAO cohort study in primary care

**DOI:** 10.3399/bjgp20X714005

**Published:** 2021-01-26

**Authors:** Emilie Ferrat, Julie Fabre, Philippe Galletout, Emmanuelle Boutin, Julien Le Breton, Vincent Renard, Paul Frappé, Sylvie Bastuji-Garin

**Affiliations:** Primary Care Department, School of Medicine, University of Paris-Est Créteil, Créteil; INSERM, IMRB, CEpiA Team (Clinical Epidemiology and Ageing), University of Paris-Est Créteil, Créteil.; Primary Care Department, School of Medicine, University of Paris-Est Créteil, Créteil.; Primary Care Department, School of Medicine, University of Paris-Est Créteil, Créteil.; University of Paris-Est Créteil, Créteil; Clinical Research Unit (URC Mondor), Assistance Publique Hôpitaux de Paris (APHP), Hôpital Henri Mondor, Créteil.; Primary Care Department, School of Medicine, University of Paris-Est Créteil, Créteil; INSERM, IMRB, CEpiA Team (Clinical Epidemiology and Ageing), University of Paris-Est Créteil, Créteil.; Primary Care Department, School of Medicine, University of Paris-Est Créteil, Créteil; INSERM, IMRB, CEpiA Team (Clinical Epidemiology and Ageing), University of Paris-Est Créteil, Créteil.; Department of General Practice, University of Saint-Etienne, Saint-Etienne.; University of Paris-Est Créteil, Créteil; Department of Public Health, APHP, Hôpital Henri Mondor, Créteil.

**Keywords:** atrial fibrillation, cohort studies, direct oral anticoagulants, prescription, primary care, public health

## Abstract

**Background:**

Direct oral anticoagulants (DOACs) account for an increasing proportion of prescriptions in patients with non-valvular atrial fibrillation (NVAF) in primary care. Inappropriate dosing of DOACs is a common problem, with under-dosing being a particular issue. However, conflicting results have been reported about the factors independently associated with inappropriate dosing.

**Aim:**

To describe inappropriate prescriptions of DOACs among patients in the CACAO French nationwide general practice cohort, and to identify the factors independently associated with inappropriate DOAC doses.

**Design and setting:**

Cross-sectional baseline analysis of the CACAO French national multicentre prospective cohort of adult patients in primary care receiving an oral anticoagulant who were recruited between April and October 2014.

**Method:**

A total of 1111 patients from the CACAO cohort who received a DOAC for NVAF were included in this study. Inappropriate prescriptions of DOACs were described (inappropriate dosage, contraindications, non-indications, interactions, and non-compliance with the precautions for use). Multivariate logistic models were used to investigate factors associated with inappropriate DOAC dosing (under-dosing and over-dosing).

**Results:**

Overall, 438 patients (39.4%) received at least one inappropriate DOAC prescription. The most common inappropriate prescription was inappropriate dosage (*n* = 374, 33.7%), particularly under-dosing (*n* = 348, 31.3%). Multivariate analysis revealed that factors independently associated with under-dosing were older age, prescription of apixaban or dabigatran, and a CHA2DS2-VASc score ≥2 vs. a score = 1. Factors with over-dosing were kidney failure, a HAS-BLED score ≥3, and older age.

**Conclusion:**

The appropriateness of DOAC prescribing for NVAF can be improved, especially in older patients, and in patients with kidney failure, a higher risk of ischaemic stroke, and/or a higher risk of bleeding. GPs have a key role in increasing the proportion of appropriate DOAC prescriptions via informational, educational, and/or management strategies.

## INTRODUCTION

Non-valvular atrial fibrillation (NVAF) is a common cardiac rhythm disorder that constitutes a significant risk factor for ischaemic stroke. One in four middle-aged adults in Europe and the US develop AF.^1^ By 2030, 14 to 17 million people in Europe are predicted to have AF, with 120 000 to 215 000 new cases per year.^1^

Oral anticoagulants are commonly prescribed for an indication of AF in ambulatory care. However, these drugs constitute the leading cause of emergency department admissions for bleeding.^2^ In a recent French cohort study, 6% of the participants taking anticoagulants experienced one or more bleeding events.^3^ Anticoagulant prescription patterns are changing with direct oral anticoagulants (DOACs), such as rivaroxaban, apixaban, dabigatran, and edoxaban, which are direct factor Xa inhibitors, being used instead of vitamin K antagonists (VKAs) to prevent stroke in patients with AF.^1^ DOACs are also used to treat thrombosis, to prevent recurrent deep vein thrombosis/pulmonary embolism, and, in the case of rivaroxaban, to prevent atherothrombotic events in acute coronary syndrome (together with aspirin or both aspirin and clopidogrel).

Since the introduction of this drug class in France in 2009, the proportion of anticoagulant-treated patients receiving a DOAC has risen continuously, reaching 38% in 2016.^4^ A variety of DOACs are available with prescription modalities that take account of factors such as the patient’s level of adherence, age, and renal function.^1^^,^^5^

Several studies have investigated inappropriate DOAC prescriptions.^6^^–^^12^ Inappropriate dosing of DOACs, particularly under-dosing, is the most common issue, affecting between 8% and 32% of patients. However, conflicting results have been reported about the factors that are independently associated with inappropriate dosing.^8^^,^^10^^,^^11^ Moreover, only one such study has been conducted in a primary care setting, and differences in prescribing patterns from one institution to another mean that its results cannot necessarily be generalised.^6^

The Comparison of Accidents and Their Circumstances with Oral Anticoagulants (CACAO) cohort is a French nationwide general practice cohort of patients who receive oral anticoagulants.^13^ The study’s primary objective was to determine whether mandatory data for the safe monitoring of oral anticoagulants are present in GPs’ records.^13^

The aims of this ancillary study were to describe the distribution of inappropriate prescriptions of DOACs among patients with NVAF in the CACAO cohort, and to investigate the factors independently associated with inappropriate DOAC dosing.

**Table:** How this fits in

Inappropriate dosing of direct oral anticoagulants (DOACs) (especially under-dosing) is the most common issue with DOACs prescription. However, conflicting results have been reported with regard to factors independently associated with inappropriate dosing. This study found that 40% of patients with non-valvular atrial fibrillation received at least one inappropriate prescription of a direct oral anticoagulant in primary care, with inappropriate dosage most common, particularly under-dosing. Factors independently associated with under-dosing were older age, prescription of dabigatran or apixaban, and a higher thromboembolism (CHA_2_DS_2_-VASc) score. Primary care physicians have a key role in increasing the proportion of appropriate prescriptions of DOACs.

## METHOD

### Setting design and participants

The CACAO study was a French nationwide multicentre prospective cohort of consecutive ambulatory patients receiving an oral anticoagulant in general practice who were recruited between April and October 2014 (see Supplementary Table S1 for details of patient characteristics). The study’s 463 GP investigators were located in 290 different rural and urban areas, and in 47 different counties throughout France. The study had two phases: an initial cross-sectional phase that examined safety data from medical records at inclusion, followed by a standard, 12-month longitudinal phase during which the efficacy and safety of VKAs versus DOACs were assessed.^13^ The CACAO study’s main inclusion criteria were age ≥18 years, prescription of a VKA or a DOAC, and a consultation (for whatever reason) with a GP investigator during the study period. Patients given injectable anticoagulants and those <18 years were not included. Patients were included in the present ancillary study if they were taking a DOAC for NVAF at inclusion (see Supplementary Figure S1 for details).

### Data collection

#### General assessment

For the purposes of the present study, only baseline data were considered. Using an electronic case report form, GPs collected data anonymously on demographics, personal medical history, current medications (from the French National Medicines Agency’s list),^14^ items from CHA_2_DS_2_-VASc and HAS-BLED (scores were calculated subsequently),^15^^,^^16^ and laboratory tests (renal and hepatic function tests, and coagulation assays). No biological samples were collected specifically for this study.

#### Outcomes

Various inappropriate DOAC prescriptions were assessed, which had been predefined by an expert group (a professor of therapeutics, five GPs, and an epidemiologist) on the basis of the summaries of product characteristics from the European Medicines Agency (see Supplementary Table S2 for details).^17^^–^^19^ These situations involved inappropriate dose prescription (under-dosing or over-dosing); prescriptions that were contraindicated or not recommended (because of a comorbidity or a concomitant medication); non-indication (a CHA_2_DS_2_-VASc <1); an at-risk interaction (antiplatelets or other reasons); and non-compliance with the precautions for use (concomitant treatments or comorbidities), that is, clinician prescribed the DOAC against the precautions for use. The reference category was normal dosage.

#### Potential factors associated with inappropriate prescriptions

Potential GP-related associated factors were age, sex, and practice environment (urban versus rural). Potential patient-related associated factors were age, sex, the type of DOAC prescribed, the CHA_2_DS_2_-VASc score (the risk per year of stroke in patients with AF), the HAS-BLED score (the risk per year of major bleeding in patients with AF), duration of prescription, the specialty of the physician having first prescribed the DOAC, other oral anticoagulants used before the current prescription, independence for DOAC administration, body mass index, current pregnancy and/or breastfeeding, concomitant antiplatelet treatment, smoking status, alcohol consumption, and associated comorbidities such as kidney failure (calculated by estimated creatinine clearance rate using the Cockcroft–Gault equation), a personal history of hypertension, symptomatic heart failure, cancer treatment in the past 6 months, diabetes mellitus, coronary heart disease and/or myocardial infarction, stroke and/or transient ischaemic attack, aortic and/or peripheral arterial disease, a personal history of haemorrhage requiring hospitalisation, deep-vein thrombosis and/or pulmonary embolism, chronic dialysis and/or kidney transplantation, and liver cirrhosis (see Supplementary Table S1 for details).

### Statistical analysis

Qualitative variables were described as number (%), and quantitative variables were described as mean (standard deviation) or median (interquartile range [IQR]) depending on their distribution. The characteristics of the GPs and patients, and the inappropriate DOAC prescription, were first described, and the factors associated with the most prevalent type of inappropriate prescription were searched for. Univariable analyses involved χ^2^ test or Fisher’s exact test for categorical variables, and Student’s *t*-test or Kruskal–Wallis test for continuous variables, as appropriate. Variables having a *P*-value <0.2 were selected for multivariable analysis and the estimation of unadjusted odds ratios (ORs). Confounders and interactions were tested in bivariate models. To avoid the introduction of highly correlated variables (such as CHA_2_DS_2_-VASc with HAS-BLED and age; or HAS-BLED with renal function), several different logistic regression models were built. A logistic model was used, because no effect of GPs was observed in an empty multilevel model. Adjusted ORs (95% confidence intervals) were estimated using multinomial logistic or exact logistic regression models. The reference category was the appropriate DOAC dose. Rivaroxaban was chosen as the reference category for the ‘DOAC molecule’ factor because it was the most prescribed drug and thus facilitated the interpretation of ORs. Goodness of fit was assessed using the Akaïke and Bayesian information criteria (lowest value = best fit). All tests were two-sided, and the threshold for statistical significance was set to *P*≤0.05. Analyses were performed with Stata software (version 15).

## RESULTS

### Study population and inappropriate direct oral anticoagulant prescriptions

The main characteristics of the 1111 patients analysed and the 112 investigating GPs are summarised in Supplementary Table S1. The median patient age was 76 years (IQR 68–82 years), 47% of the patients were female (*n* = 524), 90% had a CHA_2_DS_2_-VASc score ≥2 (*n* = 1001), 39% had a HAS-BLED score ≥3 (*n* = 437), and 54% had received a DOAC for >1 year (*n* = 604). Rivaroxaban was the most commonly prescribed DOAC (received by 50% of patients, *n* = 561). In total, 438 patients (39.4%) had received at least one inappropriate prescription. The most common inappropriate prescription was inappropriate dosage (*n* = 374, 33.7%), in particular, under-dosing (*n* = 348, 31.3%) (see Supplementary Table S1 for details).

### Factors associated with inappropriate direct oral anticoagulant dosage

In univariate analysis (see Supplementary Table S3 for details), the factors associated with inappropriate dosage were older patient age (for under- and over-dosing), prescription of apixaban or dabigatran (for under-dosing), a higher CHA_2_DS_2_-VASc score (for under-dosing), a higher HAS-BLED score (for over-dosing), and kidney failure (for over-dosing). The proportion of appropriate prescriptions was higher for rivaroxaban (74%) than for dabigatran (61%) or apixaban (45%); *P*<0.001 for both (see Supplementary Figure S2 for details).

In a multivariable model including age, the factors independently associated with under-dosing (relative to appropriate dosing) were older age, prescription of apixaban or dabigatran, and CHA_2_DS_2_-VASc score ≥2 (Table 1 and Supplementary Table S4).

**Table 1.** Multivariate multinomial logistic regression of factors associated with inappropriate dosing of direct oral anticoagulants in patients with atrial fibrillation (*n* = 1020)^a^

**Model including age**	**Adjusted OR (95% CI) for under-dosing**	***P*-value^b^**	**Adjusted OR (95% CI) for over-dosing**	***P*-value^b^**
**Patient age (years)**	1.03 (1.02 to 1.05)	<0.001	1.05 (0.99 to 1.10)	0.084

**DOAC molecule**		<0.001		0.223
Apixaban	3.93 (2.29 to 6.74)		3.14 (0.82 to 12.06)	
Dabigatran	1.55 (1.17 to 2.06)		1.02 (0.43 to 2.42)	
Rivaroxaban	1 (ref)		1 (ref)	

**Kidney function (Cockcroft–Gault) (ml/min)**		0.002		0.010
Normal, ≥60	1 (ref)		1 (ref)	
Moderate/severe/terminal failure, <60	0.59 (0.42 to 0.83)		3.28 (1.34 to 8.08)	

a Akaike information criterion = 1455.14; Bayesian information criterion =1504.42.

b *Adjusted* P*-value for all the reported variables, obtained from the Wald test using multinomial multivariate logistic regression. CI = confidence interval. DOAC = direct oral anticoagulant. OR = odds ratio.*

Factors independently associated with over-dosing, were kidney failure, a HAS-BLED score ≥3, and older age (Table 1) (see Supplementary Table S5 for more details).

## DISCUSSION

### Summary

In this large, prospective, nationwide cohort study conducted in general practice, 39% of the patients received at least one inappropriate DOAC prescription. The main type of inappropriate prescription was inappropriate dosing (33.7% of patients), most frequently under-dosing (31.3%). In a multinomial multivariable analysis, the factors independently associated with DOAC under-dosing (versus appropriate dosing) were older age, prescription of dabigatran or apixaban, and a higher CHA_2_DS_2_-VASc score. Factors independently associated with over-dosing were kidney failure, a HAS-BLED score ≥3, and older age.

### Strengths and limitations

The study’s multicentre design and the large sample size may mean that the results can be generalised reliably. Moreover, only one other study has focused exclusively on primary care, despite the fact that GPs are closely involved in the management of patients with chronic conditions who take oral anticoagulants.^6^ Given that the investigator was the patient’s family physician in 95% of cases (data not shown), the study data were easy to access by using a questionnaire. However, the data were declarative and were not checked objectively, therefore representing a potential source of measurement bias. It is also possible that GPs who agreed to participate in the study were more motivated by issues such as continuing medical education, patient education, and/or anticoagulants than the average GP.

The GPs’ records may not have included all the factors that influence decisions about DOAC dosing, including patient preferences and values, and plans for imminent cardioversions. The HAS-BLED and CHA_2_DS_2_-VASc scores were assessed a posteriori. Therefore, it was not possible to tell whether prescribers knew of these scores and took them into account when prescribing DOACs. Other confounders were also included, such as a personal history of haemorrhage requiring hospitalisation (Supplementary Table S1).

Although the concomitant prescription of DOAC and aspirin is not recommended, rivaroxaban and low-dose aspirin can be given concurrently in acute coronary syndrome and AF. However, the dose of aspirin prescribed was not recorded in the study database. Finally, the absence of longitudinal data on inappropriate prescriptions constitutes a limitation for the present analysis, but this was not the main objective of the CACAO cohort study.^3^

### Comparison with existing literature

In line with the present study’s findings, inappropriate dosing of DOAC, especially under-dosing, is usually the most prevalent issue, ranging from 7.7%^6^ to 32%^8^ in previous studies.^6^^–^^12^ However, the incidence of under-dosing observed in the current study (31.3%) was higher than in the literature. For example, the corresponding values were 7.2%, 9.4%, and 18% in the Canadian Primary Care cohort,^6^ the ORBIT-AF II Registry,^11^ and the FANTASIIA Registry,^8^ respectively. This difference might be explained by the characteristics of the study populations. In the current study, patients were older (mean age 76; standard deviation 71–75 in other studies) and more likely to have comorbidities and/or frailty factors, such as kidney failure and higher CHA_2_DS_2_-VASc scores.^6^^,^^8^^,^^11^

The most prescribed drug was rivaroxaban in the current study, as well as in the Canadian cohort^6^ and the ORBIT-AF II Registry^11^ (50%, 57%, and 54%, respectively), whereas dabigatran was the most prescribed drug in the FANTASIIA study (50%).^8^

The literature on factors associated with inappropriate dosing is contradictory.^6^^–^^12^ Older age^9^^–^^11^ and a higher CHA_2_DS_2_-VASc score^10^^,^^11^ were also factors associated with under-dosing in other studies. The fact that a higher CHA_2_DS_2_-VASc score (that is, higher risk of ischaemic stroke) was associated with under-dosing might reflect a degree of frailty among patients and the fear of over-dosing among prescribers. Similarly, the FANTASIIA study found that dabigatran was associated with under-dosing,^8^ and the ORBIT-AF II study found the same association for apixaban.^11^ In contrast with the literature on comorbidity, heart failure was not found to be a significant factor in the current study.^6^

Few studies have found that kidney failure is associated with over-dosing.^9^^,^^11^ This finding conflicts with previous reports,^6^^,^^8^^,^^10^ and might be due to the higher incidence of kidney failure in the current study’s population. However, this observation suggests that physicians might not adjust the dose level according to kidney function (possibly because of a lack of awareness or a lack of laboratory data); or perhaps they adapt the dose using the Modification of Diet in Renal Disease equation or another equation that gives better renal scores than the Cockcroft–Gault equation; or they might not have an up-to-date record of the patient’s body weight.

Other similar factors associated with over-dosing in the literature include a higher bleeding score and older age.^9^^,^^11^ However, bleeding scores (ORBIT and/or HAS-BLED) may also reflect comorbidities and/or frailty, such as older age and kidney failure, suggesting that GPs do not follow the guidelines on DOAC prescriptions.

The only other primary care study, which was carried out in Canada, reported a lower incidence of inappropriate DOAC prescriptions.^6^ Although this difference may be explained, at least in part, by the characteristics of the respective study populations (with more kidney failure and higher CHA_2_DS_2_-VASc scores in the current study), dabigatran was also less frequently prescribed in the Canadian study (34% of patients) than in the present study (43%). Moreover, around 20% of the participating physicians in the Canadian cohort were in academic practice, which may explain the lowest inappropriate prescription rate, that is, because these 20% would be less likely to make inappropriate prescriptions.

Most of the patients in the current study were first prescribed an oral anticoagulant by a cardiologist (77%); this might result in therapeutic inertia, with GPs reluctant to modify another physician’s prescription.

### Implications for practice

It is well established that higher-than-recommended dose levels of DOACs are associated with elevated all-cause mortality, and under-dosing is associated with more frequent hospitalisation for cardiovascular problems.^11^ However, some studies of off-label prescriptions have reported that stroke severity and clinical outcomes are no worse in patients with under-dosed DOACs than in patients on the recommended dose.^10^^,^^20^

In the present cohort of patients managed in primary care, most DOAC prescriptions were for the recommended doses. However, the appropriateness of DOAC prescribing can be improved for a third of patients, especially in older individuals, those with kidney failure, a higher risk of ischaemic stroke, and/or higher risk for bleeding. Cardiologists and GPs have a key role in increasing the proportion of appropriate DOAC prescriptions via informational, educational, and/or management strategies.
